# Identification of Differentially Expressed Proteins in Sugarcane in Response to Infection by *Xanthomonas albilineans* Using iTRAQ Quantitative Proteomics

**DOI:** 10.3390/microorganisms8010076

**Published:** 2020-01-03

**Authors:** Jian-Yu Meng, Mbuya Sylvain Ntambo, Philippe C. Rott, Hua-Ying Fu, Mei-Ting Huang, Hui-Li Zhang, San-Ji Gao

**Affiliations:** 1 National Engineering Research Center for Sugarcane, Fujian Agriculture and Forestry University, Fuzhou 350002, Fujian, Chinantambos@africau.edu (M.S.N.); hmt159379@163.com (M.-T.H.);; 2 CIRAD, UMR BGPI, F-34398 Montpellier, France, and BGPI, Univ Montpellier, CIRAD, INRA, Montpellier SupAgro, F-34398 Montpellier, France; philippe.rott@cirad.fr

**Keywords:** *Saccharum* spp., leaf scald, *Xanthomonas albilineans*, comparative proteomics, iTRAQ, disease resistance

## Abstract

Sugarcane can suffer severe yield losses when affected by leaf scald, a disease caused by *Xanthomonas albilineans*. This bacterial pathogen colonizes the vascular system of sugarcane, which can result in reduced plant growth and plant death. In order to better understand the molecular mechanisms involved in the resistance of sugarcane to leaf scald, a comparative proteomic study was performed with two sugarcane cultivars inoculated with *X. albilineans*: one resistant (LCP 85-384) and one susceptible (ROC20) to leaf scald. The iTRAQ (isobaric tags for relative and absolute quantification) approach at 0 and 48 h post-inoculation (hpi) was used to identify and annotate differentially expressed proteins (DEPs). A total of 4295 proteins were associated with 1099 gene ontology (GO) terms by GO analysis. Among those, 285 were DEPs during *X. albilineans* infection in cultivars LCP 85-384 and ROC20. One hundred seventy-two DEPs were identified in resistant cultivar LCP 85-384, and 113 of these proteins were upregulated and 59 were downregulated. One hundred ninety-two DEPs were found in susceptible cultivar ROC20 and half of these (92) were upregulated, whereas the other half corresponded to downregulated proteins. The significantly upregulated DEPs in LCP 85-384 were involved in metabolic pathways, the biosynthesis of secondary metabolites, and the phenylpropanoid biosynthesis pathway. Additionally, the expression of seven candidate genes related to photosynthesis and glycolytic pathways, plant innate immune system, glycosylation process, plant cytochrome P450, and non-specific lipid transfer protein was verified based on transcription levels in sugarcane during infection by *X. albilineans.* Our findings shed new light on the differential expression of proteins in sugarcane cultivars in response to infection by *X. albilineans*. The identification of these genes provides important information for sugarcane variety improvement programs using molecular breeding strategies.

## 1. Introduction

Sugarcane (*Saccharum* spp. hybrids) is an important food and bioenergy source and a significant component of the economy in more than 100 countries in the tropics and subtropics [1]. Commercial sugarcane cultivars (2*n* = 100–130) have a highly polyploid, aneuploid, heterozygous, and interspecific genome. This genome is composed of about 80% of *S. officinarum* (2*n* = 80) chromosomes, 10–15% of *S. spontaneum* (2*n* = 40–128) chromosomes, and 5–10% recombinant chromosomes between those two progenitors [2,3], thus providing major challenges for sugarcane omics researches [4]. A BAC (bacterial artificial chromosome)-based monoploid genome sequence of cultivar R570 [5] and a genome sequence of haploid *S. spontaneum* clone AP85–441 were recently reported [3]. These two genome sequences included 25,316 and 35,525 protein-coding genes, respectively. A polyploid sugarcane genome sequence of cultivar SP80–3280 from Brazil was also assembled, and this genome was composed of a gene space of 373,869 putative genes [6]. These data provide important reference sequence information in the post genomics era [4].

Because sugarcane is vegetatively propagated, the burden of certain viral and bacterial pathogens can gradually increase and result in the degeneration of sugarcane cultivars [7,8]. Leaf scald caused by *Xanthomonas albilineans* has been a destructive sugarcane disease in most sugarcane growing countries [9,10,11]. This disease was limited to a few locations of China in the early 1980s, but it recently spread to most sugarcane planting provinces of the country [10,11,12]. Sugarcane infection by *X. albilineans* can result in high losses of cane tonnage and reduced juice quality in susceptible varieties. Death of stalks and poor ratooning leads to removal of these varieties from commercial production [9]. *X. albilineans* multiplies in the xylem and colonizes the entire sugarcane plant. Infected plants of susceptible cultivars display symptoms such as white, narrow, and sharply defined leaf stripes to complete necrosis and wilting of leaves, leading to plant death [13,14]. Management of leaf scald includes planting pathogen-free seed cane and growing resistant cultivars [9].

The proteomic approach is a powerful tool for the identification of the functions of proteins expressed during plant-pathogen interactions, and therefore for a better understanding of plant immunity [15]. Investigating changes in the plant proteome, in contrast to the transcriptome, allows identification of direct effectors of plant stress responses [16]. During the last decades, the most frequently used proteomic approach was the two-dimensional gel (2-DE) technique, where differentially expressed spots were excised and analyzed by diverse mass spectrometry (MS) methods [15,17]. More advanced protein quantification techniques have been developed using tandem mass tags (TMTs) and isobaric tags for relative and absolute quantification (iTRAQ), resulting in more precise, accurate, and reproducible measurements [18]. The amine specificity of iTRAQ reagents makes most peptides in a sample amenable to this labeling strategy with no loss of information from samples involving post-translational modifications, thus providing extra statistical validation within any given experiment [19]. Recently, TMT/iTRAQ-based quantitative proteomics has been used for proteomic identification and quantification in sugarcane developmental processes and response to abiotic and biotic stresses [1].

iTRAQ quantitative proteomics has been applied to understand strategies (such as suppress/evade defense mechanisms) during the interactions between sugarcane and *Sporisorium scitamineum*. In a first study, 273 and 341 differentially expressed proteins (DEPs) were identified in two sugarcane cultivars, “Yacheng05–179” (smut-resistant) and “ROC22” (smut-susceptible) [20]. Several of these DEPs were found in the resistant cultivar such as β-1,3-glucanase, endo-1,4-β-xylanase, heat shock proteins, peroxidase, pathogenesis-related protein 1 (PR1), and lectins, thus suggesting that they may play a role in sugarcane smut resistance [20]. In another study, 209 and 125 DEPs were identified in the smut resistant cultivar GT29 and the smut susceptible cultivar Yacheng 71–374, respectively [21]. The photosynthesis pathway, reactive oxygen species (ROS), abscisic acid (ABA), and calcium signal pathway related proteins were upregulated in both cultivars. Furthermore, more DEPs were upregulated in GT29 than in Yacheng 71–374, suggesting that these DEPs may be involved in resistance to the sugarcane smut pathogen.

The objective of this study was to determine quantitative proteome changes in sugarcane cultivars LCP 85–384 (resistant to leaf scald) and ROC20 (susceptible to leaf scald) using a high-throughput iTRAQ-based technique. The identification of DEPs between these two cultivars after inoculation with *X. albilineans* provided information regarding the molecular mechanisms involved in resistance of sugarcane to leaf scald. These data are also first clues for molecular breeding of sugarcane for resistance to this disease.

## 2. Materials and Methods

### 2.1. Plant Growth and Inoculation with X. albilineans

Two sugarcane cultivars differing in resistance to leaf scald, LCP 85–384 (resistant) and ROC20 (susceptible) were used for inoculation with *X. albilineans*. LCP 85–384 originated from the Louisiana State University Agricultural Center, Sugar Research Station (St. Gabriel, LA, USA) [22] and ROC20 was provided by the Taiwan Sugar Corporation (Taiwan, China) [23]. Young plants of the two cultivars were grown in a climatic chamber at 28 °C with 60% humidity, and a 16/8 h (light/dark) photoperiod. At the 3‒5 leaf stage, and when the plant height was approximately 15‒20 cm, they were inoculated with *X. albilineans* strain Xa-FJ1 by cutting leaf blades at mid-length with sterilized scissors previously dipped in a bacterial suspension of 10^8^ CFU/mL of *X. albilineans* [10]. Control plants were inoculated with sterile XAS liquid medium [24]. The leaf samples used to extract total proteins for iTRAQ analysis were collected at 0 post-inoculation (hpi) (named R0_CK, and S0_CK for the resistant and susceptible cultivar, respectively) and 48 hpi (named R48_Xa, and S48_Xa for the resistant and susceptible cultivar, respectively). At each sampling time point, leaf tissue from six plants of each genotype were collected and then divided into three aliquots. Thus, a total of 12 samples were immediately snap-frozen in liquid nitrogen after sampling and stored at −80 °C until protein extraction and iTRAQ analysis.

### 2.2. Quantification of Populations of X. albilineans in Inoculated Sugarcane Leaves

Population size of *X. albilineans* was determined in inoculated plants at 0 and 48 hpi using a quantitative PCR (qPCR) assay developed by Garces et al. [25]. Briefly, leaf sampling was identical to those for iTRAQ analysis and then total genomic DNA was extracted using the standard CTAB protocol [10]. One microliter (µL) of total leaf DNA (100 ng/µL) and serial 10-fold dilutions (10^7^–10 copies/µL) of pMD-albI plasmid were used as qPCR templates [24]. Total DNA of a disease-free sugarcane leaf and sterile distilled water were used as negative and blank controls, respectively. Three biological replicates and three technical replicates were used for all the samples.

### 2.3. Total Protein Extraction and Peptide Preparation

To extract total proteins, frozen samples were individually ground into powder in a pre-chilled mortar with liquid nitrogen. The powder was mixed with lysis buffer containing 50 mM Tris-HCl (pH 8), 8 M Urea and 0.2% SDS. The homogenate was incubated with an ultrasonic homogenizer (JY92-IIDN, Ningbo, China) (power 150 W) on ice for 5 min and then centrifuged at 12,000× *g* for 15 min at 4 °C The ~700 µL supernatant was transferred to a new 1.5 mL centrifuge tube. Then, 7 µL 2 mM dithiothreitol was added and samples were incubated at 56 °C for 1 h, followed by addition of sufficient iodacetic acid to the sample and an additional incubation for 1 h at 25 °C in the dark. Cold acetone (four-fold volume of supernatant) was added to the sample and vortexed vigorously for ~10 s before placing samples overnight at −20 °C. The samples were centrifuged at 12,000× *g* for 15 min at 4 °C and the pellets were washed twice with cold acetone. Finally, the pellets were dissolved using dissolution buffer containing 0.1 M triethylammonium bicarbonate (TEAB, pH 8.5) and 8 M urea. Protein concentration was determined with the Bradford assay [26]. The supernatant of each sample containing precisely 0.1 mg of protein was digested with Tripsin Gold (Promega, Madison, WI, USA) at 37 °C for 16 h. The proteins were dried by vacuum centrifugation at 1000 rpm for 2 h after removal of the urea using a C18 desalting cartridge (3M Corporation, Saint Paul, MI, USA).

### 2.4. iTRAQ Labeling of Peptides

The desalted peptides were labeled with iTRAQ reagents (iTRAQ^®^ Reagent-8PLEX Multiplex Kit, Sigma-Aldrich, Shanghai, China), following the manufacturer’s instructions. For each 0.1 mg of peptide, 1 unit of labeling reagent was used. Peptides were dissolved in 20 µL of 0.5 M TEAB and the labeling reagent was added to 70 µL of isopropanol. After incubation for 1 h, the reaction was stopped with 50 mM Tris-HCl (pH 8.0). Differentially labeled peptides were mixed equally and then desalted using peptide desalting spin columns (89852; ThermoFisher Scientific, Waltham, MA, USA).

### 2.5. High-Performance Liquid Chromatography (HPLC) Fractionation

One mL TMT-labeled peptide mix was fractionated in a Waters BEH C18 column (4.6 mm × 250 mm, 5 µm) on a Rigol L3000 HPLC (Changping, Beijing, China) operating at 1 mL/min. The column oven temperature was set at 50 °C. Mobile phases A (2% acetonitrile, adjusted to pH 10.0 with ammonium hydroxide) and B (98% acetonitrile, adjusted to pH 10.0 with ammonium hydroxide) were used to obtain a gradient elution. The solvent gradient was set as follows: 3% B, 5 min; 3–8% B, 0.1 min; 8–18% B, 11.9 min; 18–32% B, 11 min; 32–45% B, 7 min; 45–80% B, 5 min; 80–5%, 0.1 min; 5% B, 6.9 min. The tryptic peptides were monitored under UV light and 214 nm wavelength. Eluent was collected every minute and then merged to 10 fractions. The samples were vacuum-dried and reconstituted in 0.1% (*v*/*v*) aqueous formic acid (FA) in water for subsequent analyses. Three biological replicates were prepared for all the samples.

### 2.6. Liquid Chromatography Mass Spectrometry (LC-MS/MS) Analysis

Shotgun proteomics analyses were performed using an EASY-nLC^TM^ 1200 UHPLC system (Thermo Fisher Scientific, Waltham, MA, USA) coupled to an Orbitrap Q Exactive HF-X mass spectrometer (ThermoFisher, Scientific, Waltham, MA, USA) operating in the data-dependent acquisition (DDA) mode. Two micrograms of total peptides reconstituted in 0.1% (*v*/*v*) FA were injected into an Accelaim PepMap100 C18 Nano-Trap column (2 cm × 150 µm, 5 µm). Peptides were separated on a Reprosil-Pur 120 C18-AQ analytical column (15 cm × 150 µm, 1.9 µm), using a 60 min linear gradient from 5 to 100% eluent B (0.1% FA in 80% acetonitrile (CAN)) in eluent A (0.1% FA in H_2_O) at a flow rate of 600 nL/min. The solvent gradient was set as follows: 6−12% B, 2 min; and 12−35% B, 50 min; 35−50% B, 2 min; 50−100% B, 1 min; and 100% B, 5 min. Three biological replicates were prepared for all the samples.

For DDA, the Q-Exactive HF-X mass spectrometer was operated in positive polarity mode with a spray voltage of 2.3 kV and a capillary temperature of 320 °C. Full MS scans from 350 to 1500 *m/z* were acquired at a resolution of 60,000 (at 200 *m/z*) with an automatic gain control (AGC) target value of 3 × 10^6^ and a maximum ion injection time of 20 milliseconds (ms). From the full MS scan, a maximum number of 40 of the most abundant precursor ions were selected for higher-energy collisional dissociation (HCD) fragment analysis at a resolution of 15,000 (at 200 *m/z*) with an automatic gain control (AGC) target value of 1 × 10^5^, a maximum ions injection time of 45 ms, a normalized collision energy of 32%, an intensity threshold of 8.3 × 10^3^, and the dynamic exclusion parameter set at 60 s.

### 2.7. Data Quality Control

The raw data obtained from MS detection were uploaded directly into proteome discovery v. 2.2 (Thermo Fisher Scientific, Waltham, MA, USA) software for database retrieval, peptide mapping and protein quantification. The retrieved results were filtered using proteome Discoverer v. 2.2. Peptide Spectrum Matches (PSMs) with 95% confidence intervals. Proteins containing at least one unique peptide fragment were considered reliable. The reliable PSMs and proteins were verified with other reliable proteins. Peptide fragments and proteins with false discovery rates (FDR) of >5% were excluded. The protein sequences of sugarcane infected by *X. albilineans* were deposited into the United States National Center for Biotechnology Information (NCBI) SRA database under accession number PXD015930.

### 2.8. Database Search and Quantitative Proteomic Analysis

The gene ontology (GO) (http://www.geneontology.org), KEGG (http://www.genome.jp/kegg/), cluster of orthologous groups of proteins (COG) (http://www.ncbi.nlm.nih.gov/COG/), and other databases were used to annotate the identified proteins. GO annotation included the analysis of identified proteins with InterProScan v. 5.22–61.0 (European Bioinformatics Institute, Cambridge, UK) and databases Pfam (http://pfam.sanger.ac.uk/), ProPrInt (http://crdd.osdd.net/raghava/proprint/), ProDom (http://prodom.prabi.fr/prodom/current/html/home.php), SMART (http://smart.embl.de/), ProSite (https://prosite.expasy.org/doc/PS51082) and PANTHER (http://www.pantherdb.org/). The KEGG and COG annotations were used in a BLAST comparison (E-value ≤ 10^−4^) of the verified proteins, and proteins with the highest scores were annotated. InterPro (IPR) also included the use of InterProScan software [27] with the Pfam, ProDom, and SMART domain databases. IPR was conducted with unknown proteins using their pattern structure or characteristics.

Proteome Discoverer v. 2.2 software was used to acquire the relative quantitative values of the PSMs of all samples. The values were based on the peak area of the plot generated by the original spectrograph. The relative quantitative values of the unique peptide fragments determined following calibration were obtained based on the quantitative data for all unique peptide fragments of each protein. For the differential analysis of proteins, the mean quantitative value of all biological repeats of each sample was used to calculate the ratio (fold change) between two samples. To determine the statistical significance of the difference, a *T*-test was conducted on the relative quantitative value of each protein for two samples to be compared. When the fold change (FC) was ≥ 1.5 and *p* ≤ 0.05, protein expression was considered significantly increased (upregulated). When FC ≤ 0.67 and *p ≤* 0.05, protein expression was considered significantly decreased (downregulated). Among all identified DEPs, protein–protein interactions (PPIs) were predicted in silico using software STRING v11.0. (https://string-db.org/) [28]. The PPIs (more than two protein interactions) data that were highly similar to sorghum proteins were retrieved and their networks were drawn using Cytoscape V3.6.1 (http://www.cytoscape.org/).

### 2.9. Quantitative Real-Time PCR (qRT-PCR) Analysis

Total RNA was extracted with the TRIzol^®^ Kit (Invitrogen, Carlsbad, CA, USA) following the manufacturer’s recommendations. Quantitative real-time PCR (qRT-PCR) analysis of the seven candidate genes was performed with the QuantStudio^®^ Real-Time PCR system (Applied Biosystems, Foster City, CA, USA). The *GAPDH* (Glyceraldehyde 3-phosphate dehydrogenase) gene was used as the internal control. First-strand cDNA was synthesized from 1 µg of total RNA using the HiScript^®^ III RT SuperMix of the qPCR (+gDNA wiper) kit (Vazyme, Nanjing, China). The resulting cDNA was then used for the qPCR assay with the ChamQ^TM^ Universal SYBR^®^ qPCR Master Mix (Vazyme, Nanjing, China) following the manufacturer’s instructions. All the primer pairs were designed with Beacon Designer software v. 8.20 (Primer Biosoft International, Palo Alto, CA, USA) and the primer sequences for each gene were listed in Supplementary Table S1. To confirm the specificity of the product and to avoid the production of primer dimmers, a dissociation curve was designed after each qPCR run. The 2^−ΔΔCT^ method was used to analyze the relative changes in expression of each gene [29]. Three independent biological replicates (aliquots) and three replicates were set for each leaf tissue sample.

### 2.10. Statistical Analyses

The paired comparison T-test was used to determine if the differences between protein (log_2_ Fold Change) and transcriptional (2^–ΔΔCT^) data of seven candidate genes at 48 hpi were significant. A general linear model was fitted to all relative expression levels (2^–ΔΔCT^) of each gene using the one-way ANOVA procedure, and Student–Newman–Keuls (SNK) test was performed with the mean values. All the statistical analyses were conducted with SAS version 8.1 (SAS Institute, Cary, NC, USA).

## 3. Results

### 3.1. Pathogen Population Size in the Resistant and the Susceptible Sugarcane Cultivar after Inoculation with X. albilineans

The population size of *X. albilineans* was six time higher in leaf scald susceptible cultivar ROC20 (mean of 611 copies of *X. albilineans* genome/µL) than in resistant cultivar LCP 85–384 (mean of 102 copies of *X. albilineans* genome/µL) at 48 hpi. No genome of *X. albilineans* was detected in the control plants inoculated with sterile liquid medium (Ct values greater than 35).

### 3.2. Overview of Proteomic Profiling of Sugarcane Infected by X. albilineans

iTRAQ analysis revealed 574,559 and 548,154 total spectra, 21,703 and 19,350 unique peptides, and 6126 and 5463 proteins for the non-infected control plants (R0_CK and S0_CK) and the plants inoculated with *X. albilineans* (R48_Xa and S48_Xa), respectively. A total of 6891 proteins (at least one unique peptide with > 95% confidence) were identified among all proteins from all samples when screened against the *Saccharum* spp. unigene database (P101SC18020747-01) (Table 1). Among those, 4295 proteins were annotated to 1099 GO terms after GO analysis. Among the 2231 proteins annotated in the biological process category, most proteins were distributed into the oxidation-reduction process (525 proteins, 24%), the metabolic process (280 proteins, 13%), proteolysis (163 proteins, 7%), and the carbohydrate metabolic process (156 proteins, 7%). The 1015 proteins annotated in the cell component category were distributed into 10 different subcellular locations: ribosome (150 proteins, 15%), membrane (144 proteins, 14%), intracellular (140 proteins, 14%), cytoplasm (128 proteins, 13%), integral component of the membrane (100 proteins, 10%), nucleus (88 proteins, 9%), proteasome core complex (24 proteins, 2%), photosystem II (23 proteins, 2%), photosystem II oxygen evolving complex (21 proteins, 2%), and nucleosome (20 proteins, 2%) (Supplementary Figure S1). Among the 2998 proteins annotated in the molecular function category, most proteins were attributed to the following functions: ATP binding (516 proteins, 17%), protein binding (453 proteins, 15%), and nucleic acid binding (230 proteins, 8%) (Supplementary Figure S1). Using the KEGG pathway analysis, 2740 proteins were annotated in 107 pathways such as carbohydrate metabolism, translation, and amino acid metabolism, etc. (Supplementary Figure S2).

### 3.3. Identification of Differentially Expressed Proteins (DEPs) in Response to X. albilineans Infection

**Figure 1:**
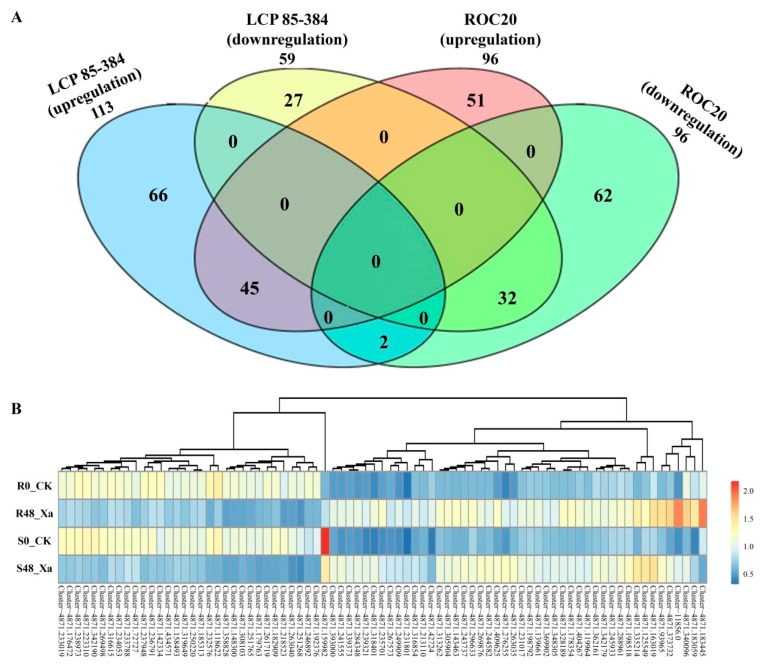
(**A**) Venn diagram of the 285 differentially expressed proteins (DEPs) and (**B**) heat map of expression changes (average of three biological replicates) of 79 common DEGs identified in two sugarcane cultivars inoculated with *Xanthomonas albilineans*. LC P85–384 is resistant to leaf scald and ROC20 is susceptible to the disease. The number of statistically significant DEPs was based on a *p* value ≤ 0.05 and a fold change of protein expression ≥ 1.5 (upregulation) or ≤ 0.67 (downregulation). The total numbers of DEPs for each treatment are shown outside the circles and the numbers of DEPs exclusively expressed in one treatment are shown in each circle. The overlapping section of two-four circles represents the common DEPs between all treatments: upregulated DEPs in R48_Xa vs. R0_CK (in blue), downregulated DEPs in R48_Xa vs. R0_CK (in yellow), upregulated DEPs in S48_Xa vs. S0_CK (in pink), downregulated DEPs in S48_Xa vs. S0_CK (in green). R0_CK = cultivar LCP85–384 inoculated with sterile liquid medium, R48_Xa = cultivar LCP85–384 inoculated with *X. albilineans*, S0_CK = cultivar ROC20 inoculated with sterile liquid medium, S48_Xa = cultivar ROC20LCP85–384 inoculated with *X. albilineans* The red scale color in (**B**) is associated with upregulation whereas the blue scale color corresponds to downregulation.

A total of 285 DEPs were identified for the two sugarcane cultivars after inoculation with *X. albilineans* and based on a *p* value ≤ 0.05 and an expression change ≥ 1.5 (up-regulation) or ≤ 0.67 (down-regulation) (Supplementary Figure S3). Among those, 164 DEPs were upregulated (Supplementary Table S2) and 123 DEPs were downregulated (Supplementary Table S3). For resistant cultivar LCP 85–384, 172 DEPs were found after inoculation with *X. albilineans* (R48_ vs. R0_CK) and among those, 113 were upregulated and 59 were downregulated. For susceptible cultivar ROC20, 192 DEPs were identified after inoculation with the pathogen (S48_Xa vs. S0_CK), and the same number of DEPs (96) was upregulated and downregulated (Figure 1A). Seventy-nine DEPs were shared by both cultivars. Among those, 45 were upregulated and 32 were downregulated, whereas two proteins (Cluster-4871.159982 and Cluster-4871.183445) were upregulated in LCP 85–384 but downregulated in ROC20 (Figure 1B).

### 3.4. Gene Annotation of DEPs

**Figure 2:**
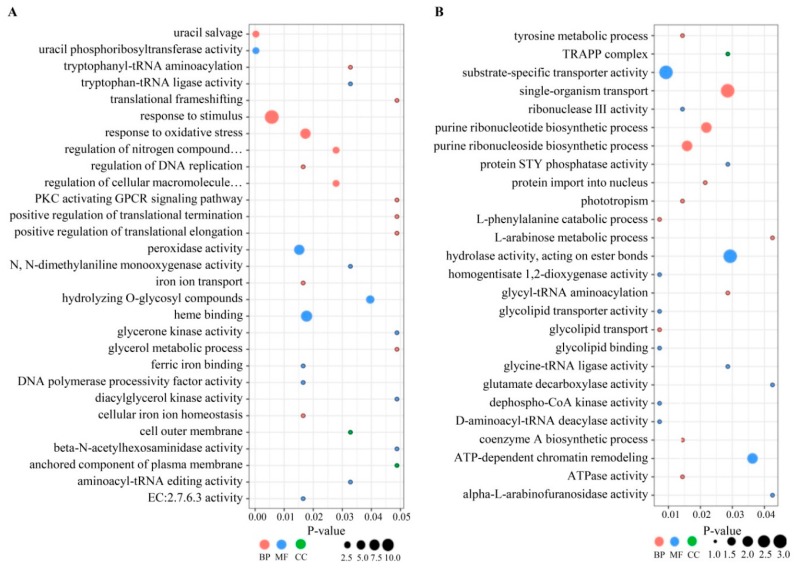
Gene ontology (GO) classification of significantly upregulated (**A**) and downregulated (**B**) differentially expressed proteins (DEPs) in sugarcane cultivar LCP 85–384 inoculated with *Xanthomonas albilineans* (R48_Xa vs. R0_CK). LCP 85–384 is resistant to leaf scald. DEPs were annotated with a GO term belonging to one of three biological process categories: biological process (BP, in pink color), molecular function (MF, in blue color), and cellular component (CC, in green color). The X-axis represents the *p*-value and Y-axis shows the GO term names.

Seventy-six of the 285 DEPs identified by GO enrichment analysis were attributed to 21 functional groups. The most significantly enriched GO terms (>5 DEPs) in the library of resistant cultivar LCP 85–384 (R48_Xa vs. R0_CK) included “response to stimulus (GO:0050896)” and “response to oxidative stress” (GO:0006979) of the biological process category, as well as “heme binding (GO:0020037)” and “peroxidase activity” (GO:0004601) of the molecular function category (Figure 2). For the library of susceptible cultivar ROC20 (S48_Xa vs. S0_CK), the most significantly enriched GO terms (>5 DEPs) were “hydrolase activity, acting on ester bonds (GO:0016788)” of the molecular function category and “cellular component organization or biogenesis (GO:0071840)” of the biological process category. The DEPs that corresponded to the most significantly enriched GO terms in LCP 85–384 were upregulated proteins, whereas those in ROC20 were downregulated proteins (Supplementary Figure S4).

### 3.5. Functional Classification of DEPs by KEGG Analysis

Most proteins identified by KEGG analysis were involved in metabolic pathways (map01100) of the two sugarcane cultivars (Supplementary Table S4). Proteins involved in biosynthesis of secondary metabolites (map01110) were significantly upregulated in resistant cultivar LCP 85–384 (Supplementary Table S4). Some amino acid metabolism pathways (map00250 and map00270), purine and pyrimidine metabolism pathways (map00230 and map00240), and amino sugar and nucleotide sugar metabolism (map00520) were significantly enriched in both cultivars (Table 2). Proteins associated with defense response pathways were essentially expressed in the leaf scald resistant cultivar (LCP 85–384) and included DEPs associated with phenylpropanoid biosynthesis pathway (map00940), ubiquitin mediated proteolysis (map04120), and glutathione metabolism (map00480) (Supplementary Table S4). Two photosynthesis-related pathways were also involved in the response of sugarcane to *X. albilineans* infection: photosynthesis pathway (ko00195) and photosynthesis-antenna proteins pathway (ko00196) (Table 2).

### 3.6. Protein–Protein Interactions (PPIs) Network Predicted in the STRING Database

**Figure 3:**
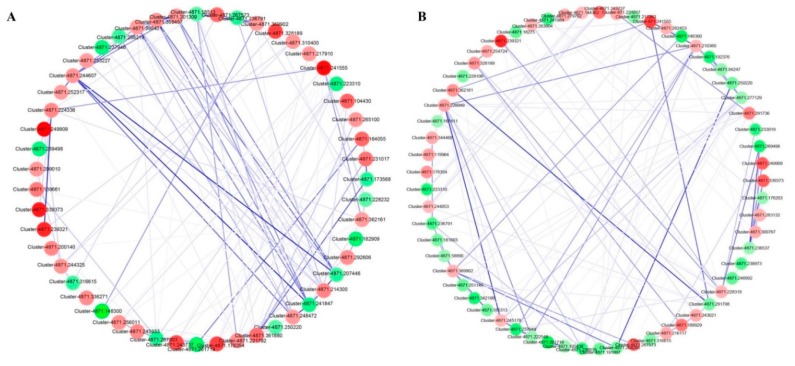
Illustration by STRING analysis of protein–protein interactions (PPIs) networks in sugarcane cultivars LCP85–384 (**A**) and ROC20 (**B**) inoculated with *X. albilineans*. Blue lines connect proteins of the PPI networks and the darker blue colors indicate higher core PPI values. The red and green colors indicate the upregulated and downregulated DEPs, respectively. Darker colors of circles are related to higher log_2_ Fold Change values of DEPs. The protein IDs were listed in Supplementary Tables S2 and S3.

The STRING database is a large repository of protein–protein interaction networks, including functional interactions, regulatory interactions, and stable complexes of proteins. The PPIs of the 285 sugarcane DEPs were identified by submitting a protein query sequence in the search box of the database. Ninety-two DEPs interacted with each other in the two varieties inoculated with *X. albilineans.* Among those, 51 DEPs (36 upregulated and 15 downregulated) were found in LCP 83–384 whereas 61 DEPs (29 upregulated and 32 downregulated) were found in ROC20 (Figure 3). A majority of these proteins were enriched in the metabolic (ko01100), the biological secondary metabolic (ko01110), glutathione (ko00480) and glycolysis/gluconeogenesis pathways (ko00010).

### 3.7. Identification of Plant Defense Genes Triggered by Colonization of Sugarcane by X. albilineans

**Figure 4:**
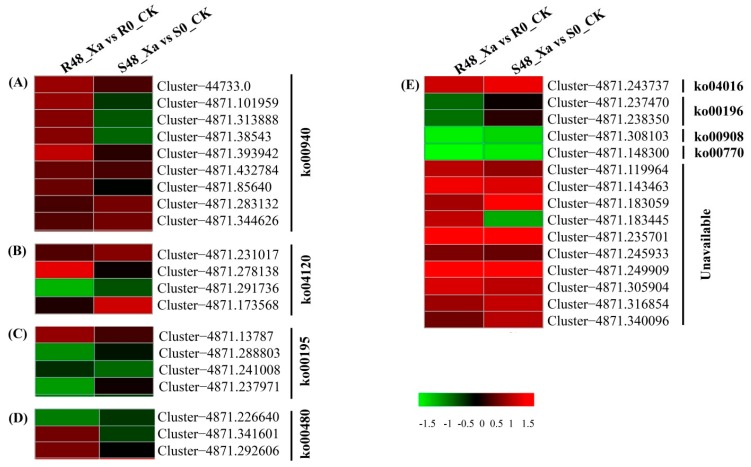
Hierarchical clustering of 35 differentially expressed proteins (DEPs) enriched in different plant-defense related pathways of two sugarcane cultivars inoculated with *Xanthomonas albilineans*. Data were recorded 48 h post inoculation: R48_Xa vs. R0_CK for LCP 85–384 (resistant to leaf scald) and S48_Xa vs. S0_CK for ROC20 (susceptible to leaf scald). (**A**) phenylpropanoid biosynthesis (ko00940); (**B**) ubiquitin mediated proteolysis (ko04120), (**C**) photosynthesis (ko00195), (**D**) glutathione metabolism (ko00480), (**E**) other pathways including MAPK signaling pathway-plant (ko04016), photosynthesis-antenna proteins (ko00196), zeatin biosynthesis (ko00908), pantothenate and CoA biosynthesis (ko00770). Colored boxes in each column represent a relative expression of protein (log_2_ Fold Change from −1.5 to +1.5).

To further characterize the DEPs attributed to defense-related pathway categories in LCP 85–384 or ROC20, 35 representative DEPs from these categories were selected for further characterization, including phenylpropanoid biosynthesis (nine DEPs), ubiquitin mediated proteolysis (four DEPs), glutathione metabolism (three DEPs), MAPK signaling pathway (one DEP), zeatin biosynthesis (one DEP), pantothenate and CoA biosynthesis (one DEP) (Supplementary Table S5). Proteins related to defense response pathways were upregulated in the two cultivars, particularly in LCP 85–384. Of nine upregulated DEPs of the phenylpropanoid biosynthesis pathway (map00940), seven and two proteins were expressed in LCP 85–384 and ROC20, respectively (Supplementary Table S5 and Figure 4). Of the four DEPs involved in ubiquitin mediated proteolysis (ko04120), one protein (ubiquitin-activating enzyme E1, Cluster-4871.278138) was upregulated whereas one protein (unidentified protein, Cluster-4871.291736) was downregulated in LCP 85–384, and two proteins (a hypothetical protein, Cluster-4871.231017 and an unidentified protein, Cluster-4871.173568) were upregulated in ROC20. In the glutathione metabolism pathway (ko00480), two proteins (unidentified protein, Cluster-4871.341601; hypothetical protein, Cluster-4871.292606) were upregulated and one protein (unknown [*Zea mays*], Cluster-4871.237470) was downregulated in LCP 85–384. The serine/threonine-protein kinase SAPK2-like isoform (Cluster-4871.243737) involved in the MAPK signaling pathway (ko04016) was upregulated, whereas the cis-zeatin O-glucosyltransferase (Cluster-4871.308103) of the zeatin biosynthesis pathway (ko00908), as well as a hypothetical protein (Cluster-4871.148300) of pantothenate and CoA biosynthesis (ko00770) pathway, were downregulated in LCP 85–384 and ROC20 (Supplementary Table S5 and Figure 4).

### 3.8. Transcript Profiling of Seven Selected Genes by qRT-PCR

**Figure 5:**
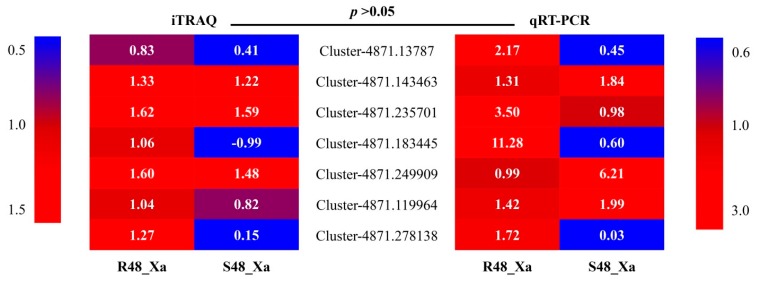
Relative expression levels (values in boxes) of seven sugarcane genes based on iTRAQ (log_2_ Fold Change) and qRT-PCR (2^–ΔΔCT^) data of two cultivars inoculated with *Xanthomonas albilineans*. Data were recorded at 48 h post inoculation: R48_Xa for leaf scald-resistant cultivar LCP 85–384 and S48_Xa for leaf scald-susceptible cultivar ROC20. The paired comparison T-test was used to determine if differences between iTRAQ (log_2_ Fold Change) and qRT-PCR (2^–ΔΔCT^) data were significant at *p* = 0.05.

Seven candidate genes were selected for further investigation of their transcript profiling because the DEPs encoded by these genes were involved in two main KEGG pathways and five important disease-resistance gene families (Supplementary Table S1). The transcription level of these seven genes was determined by qRT-PCR analysis at 0 and 48 hpi. The PCR amplification efficiency of the seven genes ranged from 98% to 101%. Relative expression between protein levels (log_2_ Fold Change) and transcriptional levels (2^–ΔΔCT^) of these genes at 48 hpi was not significantly different according to the paired comparison T-test (*p*-values ranged from 0.1678 to 0.2172) (Figure 5).

**Figure 6:**
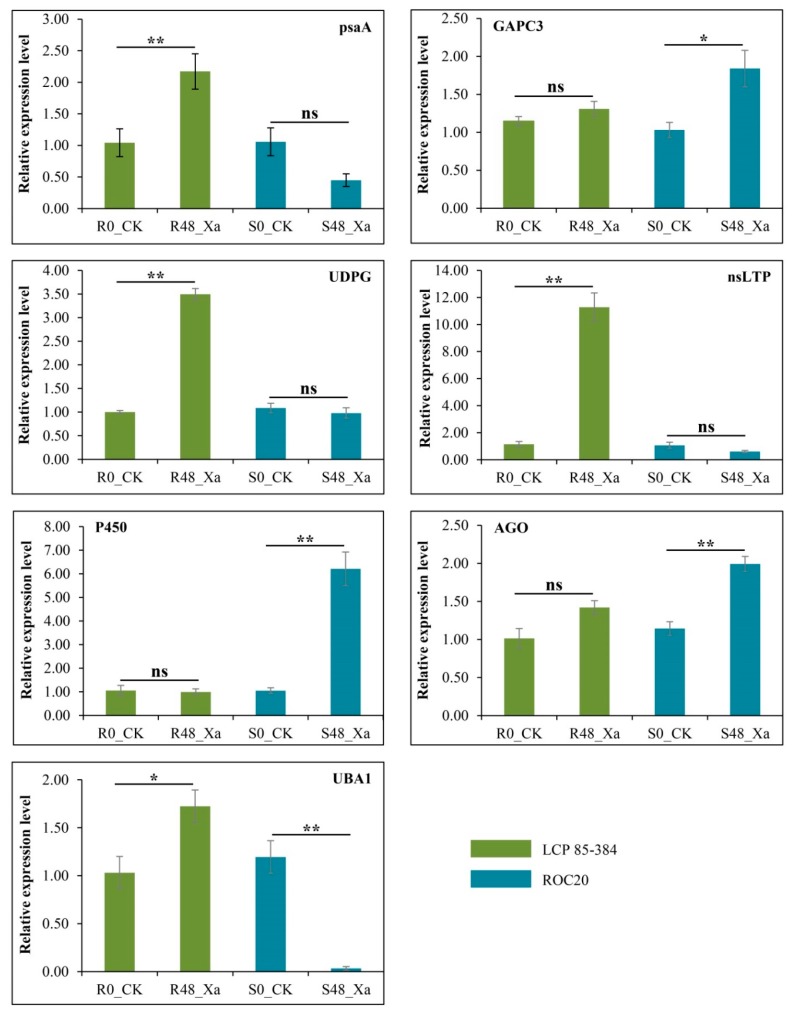
Relative expressions of seven differentially expressed proteins (DEPs) identified in leaves of two sugarcane cultivars inoculated with *Xanthomonas albilineans*. LCP 85–384 is resistant to leaf scald and ROC20 is susceptible to the disease. Gene expression was determined by qRT-PCR analysis at 0 and 48 h after plant inoculation. R0_CK (LCP 85–384) and S0_CK (ROC20) were inoculated with sterile liquid medium and R48_Xa and S48_Xa were inoculated with *X. albilineans*. Each column represents the mean value of three aliquots from six pooled plants and three technical replicates. The vertical bar at the top of each column is the standard error of the mean. Differences in gene expression for each gene and each cultivar were analyzed using the Student–Newman–Keuls test (* = *p* < 0.05; ** = *p* < 0.01). psaA = photosystem I P700 apoprotein A1 (Cluster-4871.13787), GAPC3 = Cytosolic glyceroldehyde-3-phosphate dehydrogenase (Cluster-4871.143463), UGT = UDP-glycosyltransferase (Cluster-4871.235701), nsLTPs = non-specific lipid transfer protein (Cluster-4871.183445), P450 = plant cytochrome P450 72A15 (Cluster−4871.249909), AGO = argonaute family protein (Cluster-4871.119964), UBA1 = ubiquitin-activating enzyme E1 (Cluster-4871.278138).

Four genes were highly expressed in the leaf scald resistant and susceptible cultivars as compared to the pathogen-free control plants: *GAPC3* coding for the cytosolic glyceroldehyde-3-phosphate dehydrogenase, *UGT* coding for the UDP-glycosyltransferase, *nsLTP* coding for the non-specific lipid transfer protein, and *UBA1* coding for the ubiquitin-activating enzyme E1 (Figure 6). Three genes coding respectively for the photosystem I P700 apoprotein A1 (*psaA*, Cluster-4871.13787), a non-specific lipid-transfer protein (*nsLTP*, Cluster-4871.183445), and the ubiquitin-activating enzyme E1 (*UBA1*, Cluster-4871.278138) were all downregulated in ROC20 in comparison to pathogen-free control plants. The UDP-glycosyltransferase (*UGT*, Cluster-4871.235701) and *nsLTP* gene (Cluster-4871.183445) were highly upregulated (*p* < 0.01) in resistant cultivar LCP 85–384 with 3.5- and 9.9-fold changes, respectively. The *psaA* gene was also significantly highly expressed (*p* < 0.05), with a fold change of 2.1 in LCP 85–384. The genes coding for cytochrome P450 (*P450*, Cluster-4871.249909), the cytosolic glyceroldehyde-3-phosphate dehydrogenase (*GAPC3*, Cluster-4871.143463), and the argonaute family protein (*AGO*, Cluster-4871.119964) were significantly upregulated with 5.3-, 1.8-, and 1.7-fold change, respectively, in ROC20. The *UBA1* (Cluster-4871.278138) gene was significantly upregulated with a 1.7-fold change in LCP 85–384 and was almost not expressed in ROC20 after inoculation with *X. albilineans* (Figure 6).

## 4. Discussion

### 4.1. Overall Assessment of DEPs Involved in Response to X. albilineans Infection

This comparative proteomic study using the iTRAQ technique resulted in identification of 4295 proteins involved in response of sugarcane to infection by *X. albilineans*. More than 500 of these proteins were attributed to the oxidation-reduction process (biological process category) and ATP binding (molecular function category), while most of the predicted proteins were annotated as proteins of carbohydrate metabolism, as well as translation and amino acid metabolism pathways. For the leaf scald resistant cultivar LCP 85–384, 27 DEPs were enriched in metabolic pathways and 16 DEPs were enriched in biosynthesis of secondary metabolites, indicating that metabolites play key roles in sugarcane in response to *X. albilineans* infection. Large numbers of primary and secondary metabolites play vital roles in plant defense mechanisms involving complex cascades [30,31,32]. Besides metabolic pathways and the biosynthesis of secondary metabolites, infection of sugarcane by *X. albilineans* also triggered plant-defense related pathways such as phenylpropanoid biosynthesis, ubiquitin mediated proteolysis, glutathione metabolism, and photosynthesis. PPI network analysis also indicated that these important pathways participate in sugarcane resistance and defense response during *X. albilineans* infection. Indeed, the above-mentioned pathways are commonly activated in sugarcane in response to various pathogens [1].

### 4.2. Regulation of Photosynthesis and Glycolytic Pathways of Sugarcane in Response to X. albilineans Infection

Generally, genes related to photosynthesis are downregulated as chlorotic and necrotic tissues develop during infection of plants by pathogens [33,34]. However, in our study, the *psaA1* gene (Cluster-4871.13787) was significantly upregulated in the leaf scald resistant cultivar and only slightly downregulated in the susceptible cultivar at protein and transcription levels. This suggested that stimulation of the first step of photosynthesis occurs during the early stages of expression of sugarcane lead scald resistance to allow efficient light-driven electron transport. Notably, psaA1 is one of PSI P700 apoproteins that are the primary electron donors of photosystem I (PSI) [35,36]. At the early stage (48 hpi) of plant infection by *X. albilineans*, no white-pencil lines nor chlorotic symptoms appeared on the two sugarcane cultivars. Similarly, the final receptors of electrons in light-dependent reactions (encoded by the ferredoxin [2Fe-2S] and ferredoxin-DNADP+ reductase genes) were overproduced during infection of sugarcane by *Acidovorax avenae* subsp. *avenae* [37]. Genes related to the photosynthesis-antenna proteins were also upregulated in *Cucumis sativus* against *Cucurbit chlorotic yellows virus* infection [38].

GAPC3 is one of the glyceraldehyde-3-phosphate dehydrogenase (GAPDH) gene families which play crucial roles in cellular processes [39]. In addition to being involved in glycolysis, GAPC is a phosphorylating NAD-dependent GAPDH catalyzing the conversion of glyceraldehyde-3-P (Ga3P) to 1,3-bisphosphoglycerate in the cytoplasm [40]. In our study, *GAPC3* (Cluster-4871.143463) was highly expressed in both sugarcane cultivars but was upregulated in susceptible ROC20 at both protein and transcription levels. This suggested that GAPC3 does not contribute to disease resistance but may favor progress of the pathogen during plant colonization. Overexpression of *MeGAPCs* in cassava resulted in decreased disease resistance against *X. axonopodis* pv *manihotis*, the causal agent of cassava bacterial blight. In contrast, *MeGAPCs*-silenced cassava plants by virus-induced gene silencing conferred improved disease resistance, as evidenced by physical interaction of MeGAPCs with autophagy-related protein 8b (MeATG8b) and MeATG8e, and inhibition of autophagic activity [41].

### 4.3. Activation of Plant Innate Immune Systems in Sugarcane after Inoculation with X. albilineans

Ubiquitination is one of the posttranslational protein modifications governing plant immune responses [42,43,44]. The process of ubiquitination involves covalent attachment of the highly conserved small protein ubiquitin to substrate proteins through a stepwise enzymatic cascade. The ubiquitin-activating enzyme (E1 or UBA) is one of three different catalytic enzymes that typically catalyze at the initial step of ubiquitination [45,46]. In tobacco, the expression of *NtUBA1* and *NtUBA2* was regulated in response to viral infection, wounding, and defense-related hormones [47]. In *Arabidopsis*, enzyme E1 was involved in R-protein-mediated resistance [42]. Genome-wide analysis of genes encoding core components of the ubiquitin system in soybean revealed that a large number of UBS-related genes (including E1 gene *GmUBA1*) played a role in immunity against soybean cyst nematode [48]. In our study, UBA1 was overexpressed in leaf scald resistant LCP 85–384 at protein and mRNA levels, but highly repressed in susceptible ROC20, especially at transcription level. These results indicated the *UBA1* gene plays an active role in sugarcane in response to colonization by *X. albilineans*.

RNA silencing plays a major role in regulating plant developmental processes and environmental adaptation to diverse biotic and abiotic stresses through transcriptional gene silencing (TGS) and post-transcriptional gene silencing (PTGS) [49]. In addition to a ribonuclease III-type dicer-like (DCL) enzyme and RNA-dependent RNA polymerases (RDRs), an Argonaute (AGO) protein (catalytic core in RNA-induced silencing complex (RISC) is also a core component of the RNA silencing process [50]. Of the 10 *Arabidopsis* AGO protein families, AtAGO4 participates in the repeat-associated siRNA (ra-siRNA) pathway mediating methylation of DNA repeats [51,52]. This protein is also involved in the miRNA pathway mediating gene expression, as evidenced by a subset of the miRNAs preferential association with AtAGO4 [53,54]. AtAGO4 is required for the resistance of *Arabidopsis* to *Pseudomonas syringae* and AGOs (including AGO4 protein) of *Brassica napus* are involved in plant resistance to the necrotrophic fungal pathogen *Sclerotinia sclerotiorum* [55,56]. In our study, the sugarcane *AGO* gene (a homologue of *Zea mays* AGO4, LOC100381552) was highly upregulated in the leaf scald resistant and in the susceptible cultivar, but especially in ROC20. This indicated that the AGO4 protein was activated in sugarcane during infection by *X. albilineans.* However, the molecular mechanisms involving AGO4 during the interactions between sugarcane and *X. albilineans* remain to be deciphered.

### 4.4. High Level Expression of the UDP-Glycosyltransferase in the Sugarcane Cultivar Resistant to Leaf Scald

The UDP-glycosyltransferase (UGTs; EC 2.4.1.91) catalyzes the transfer of sugar molecules to a variety of acceptor molecules, such as hormones, lipids and other small molecules [57,58]. The glycoside molecule then regulates the biological activity, water solubility and stability of receptors [57,59,60]. Up to now, 106 GT families have been identified in the carbohydrate-active enzyme database (CAZy; http://www.cazy.org/). The largest of these families is GT Family 1 that comprises a large number of UGT members [61,62]. UGTs play an important role in the regulation of plant hormone balance, detoxification of endogenous and exdogenous substances, and modification of secondary metabolites [58,60]. Recently, several studies suggested that the UDP-glycosyltransferase was positively regulated in plant species in response to pathogens. For example, expression of *TaUGT4* in wheat was higher in a Fusarium head blight (FHB) resistant cultivar than in a susceptible one after treatment with deoxynivalenol (DON) produced by *Fusarium graminearum* [63]. Wheat overexpressing TaUGT5 was also more resistant to *F. graminearum* as evidenced by reduced proliferation and destruction of plant tissue by the pathogen [64]. Gene *HvUGT-10W1* from barley conferred resistance to FHB [65]. The overexpression of the *BnUGT74B1* gene in *B. napus* increased the aliphatic and indolic glucosinolates levels by a factor 1.7 and 1.5 in leaves infected by *Sclerotinia sclerotiorum* and *Botrytis cinerea*, respectively [66]. *BrUGT74B1* was also involved in phytoalexins biosynthesis which has an important role in plant disease resistance [67].

In our study, a sugarcane UDP-glycosyltransferase (a homolog of UGT73C2 of *A. thaliana*) was highly upregulated at 48 hpi in the resistant cultivar following inoculation by *X. albilineans*, suggesting that this gene plays an important role during the defense of sugarcane against this pathogen. The receptor molecules of sugarcane involved in the glycosylation process remain to be identified. A primary response of sugarcane to *Ustilago scitaminea* infection appears to be the production of glycoproteins inhibiting germination and inducing aggregation of fungal teliospores [68].

### 4.5. Different Expression Patterns of Plant Non-Specific Lipid Transfer Proteins (nsLTPs) in the Leaf Scald Resistant and the Leaf Scald Susceptible Sugarcane Cultivar

nsLTPs are a group of small, basic proteins that are abundantly expressed in plants, having the ability to bind or transfer various types of hydrophobic molecules in vitro, such as fatty acids, fatty acyl-CoA, phospholipids, glycolipids, and cutin monomers [69,70]. nsLTPs are involved in key cellular processes such as the stabilization of membranes, cell wall organization, and signal transduction, in addition to responses to stress and developmental processes [70]. Notably, nsLTPs exhibit strong antimicrobial activity in vitro and interfere with the membrane of target organisms, thus leading to the loss of membrane integrity [70]. In our study, a sugarcane *nsLTP* gene (homolog of *Zea may* nsLTP I) was strongly overexpressed in leaf scald resistant cultivar LCP 85–384 at both protein and transcription levels, suggesting that this gene plays a positive role in resistance to *X. albilineans*. Similar observations were reported in other plants in response to infection by various pathogens. For example, several nsLTP isoforms of *Trichoderma harzianum* T39-treated grapevines increased after *Plasamopara viticola* inoculation [71]; the early expression level of the *nsLTP* gene was significantly increased in wheat infected by the rust pathogen *Puccinia triticina* before visible haustoria formation [72]. The *nsLTP* gene is also involved in the priming acquisition at the early priming stage and memory in beta-aminobutyric acid (BABA)-primed mango fruit after *Colletotrichum gleosporioides* inoculation [73]. These findings provided additional proofs of participation of nsLTPs in the defense response of plants to pathogens.

### 4.6. Upregulation of the Plant Cytochrome P450 after Colonization of Sugarcane by X. albilineans

Plant P450s participate in a large number of primary and secondary metabolisms, including the phenylpropanoid, flavonoid, cyanogenic glucoside, essential sterols and steroid hormones, and other biosynthetic pathways which are thought to convey adaptive advantages in specific ecological niches [74,75,76,77]. P450s acting on fatty acids (FA)-involved oxygenation reactions in plants is enhanced by biotic and abiotic stress at the transcriptional level [78]. For example, CYP709C1 (a P450 protein) was the first sub-terminal hydroxylase of long-chain FAs characterized in plants and its induction by methyl jasmonate resulted in plant defense reaction [79]. The P450 protein CYP74 of *A. thaliana* catalyzed the generation of oxylipins (jasmonates, aldehydes, divinyl ether, and alcohols) that acted as not only signaling molecules, but also exhibited antimicrobial and antifungal properties [80]. Oxidation of JA-isoleucine conjugate (JA-Ile) by cytochrome P450 monooxygenase is the major mechanism for turning off JA signaling [81,82]. Jasmonates are lipid-derived compounds that act as signals in plant stress responses and developmental processes [82,83].

In sugarcane, cytochrome P450 sequences have been annotated in the genome of *S. spontaneum* AP85–441 [3,84]. A sugarcane P450 protein showed interaction activities with ScMat1, a putative sugarcane transcription factor TFIIH subunit that has kinase activity [85]. Additionally, sugarcane gene *ScCPR450* was highly expressed at the mRNA level in plants under SA or PEG stresses, suggesting that this gene plays a role in the response of sugarcane to stresses [86]. In our study, expression of a sugarcane *P450* gene (homolog of *Setaria italica* P450 72A15) was increased in the leaf scald resistant and susceptible cultivars, particularly in the susceptible cultivar. A similar observation showed that *GbCYP86A1-1* positively regulated the defense of *Gossypium barbadense* against *Verticillium dahliae* by cell wall modification and the activation of immune pathways [87]. However, the precise role of P450 proteins in sugarcane in response to pathogen infection remains to be determined.

## 5. Conclusions

This study provides the first global proteomic dataset of sugarcane in response to infection by *X. albilineans.* The DEPs that were identified are predicted to be involved in metabolic pathways and biosynthesis of secondary metabolites as well as some plant-defense related pathways, such as phenylpropanoid biosynthesis and plant immune signal transduction. Seven candidate genes coding for a UDP-glycosyltransferase, a non-specific lipid-transfer protein, an acytochrome P450 protein, a cytosolic glyceroldehyde-3-phosphate dehydrogenase, an argonaute family protein, the ubiquitin-activating enzyme E1, and the photosystem I P700 apoprotein A1 (chloroplast) were activated or repressed at the transcriptional level after inoculation with the leaf scald pathogen. These genes are good candidates for further investigations of the pathways involved in the sugarcane response to infection by *X. albilineans*.

## Figures and Tables

**Table 1:** Statistics of the proteins identified by iTRAQ in two sugarcane cultivars inoculated with *Xanthomonas albilineans* (LC P85–384 resistant and ROC20 susceptible to leaf scald).

Run Name ^a^	Total Spectra	Number of Peptides	Number of Proteins
Run1	574,559	21,703	6126
Run2	548,154	19,350	5463
All			6891

^a^ Run1 included one mixed sample (reference) and six samples from plants of resistant cultivar LCP 85–384 (3 aliquots: R0_CK1, R0_CK2, R0_CK3) and susceptible cultivar ROC20 (S0_CK1, S0_CK2, S0_CK3) inoculated with pathogen-free culture medium; Run2 included one mixed sample (reference) and six samples from plants of resistant cultivar LCP 85–384 (R0_Xa1, R0_ Xa2, R0_ Xa3) and susceptible cultivar ROC20 (S0_ Xa1, S0_ Xa2, S0_ Xa3) inoculated with *X. albilineans.*

**Table 2:** Number of differentially expressed proteins (DEPs) in major KEGG pathways identified in two sugarcane cultivars LCP 85–384 (resistant to leaf scald) and ROC20 (susceptible to leaf scald) after inoculation with *Xanthomonas albilineans* ^a^.

Pathway ID	Pathway Name	R48_Xa vs. R0_CK		S48_Xa vs. S0_CK	
		Upregulation	Downregulation	Upregulation	Downregulation
ko01100	Metabolic pathways	27	0	24	11
ko01110	Biosynthesis of secondary metabolites	16	0	13	0
ko00940	Phenylpropanoid biosynthesis	7	0	2	0
ko03013	RNA transport	4	0	0	0
ko00250	Alanine, aspartate and glutamate metabolism	3	1	3	0
ko00520	Amino sugar and nucleotide sugar metabolism	3	1	2	2
ko00630	Glyoxylate and dicarboxylate metabolism	3	0	0	0
ko00480	Glutathione metabolism	2	1	0	0
ko00561	Glycerolipid metabolism	2	0	1	0
ko03420	Nucleotide excision repair	2	0	1	0
ko00511	Other glycan degradation	2	0	2	0
ko04146	Peroxisome	2	0	2	0
ko00230	Purine metabolism	2	0	3	0
ko04120	Ubiquitin mediated proteolysis	1	1	2	0
ko00650	Butanoate metabolism	1	1	1	0
ko00195	Photosynthesis	1	2	0	1
ko04075	Plant hormone signal transduction	1	0	1	0
ko00640	Propanoate metabolism	1	0	2	0
ko00280	Valine, leucine and isoleucine degradation	2	0	1	0
ko00920	Sulfur metabolism	0	2	0	3
ko03010	Ribosome	0	4	0	2
ko00270	Cysteine and methionine metabolism	0	2	0	1
ko01230	Biosynthesis of amino acids	0	0	0	3

^a^ Only showing the KEGG pathways with ≥ 3 DEPs. R48_Xa (LCP 85–384) and S48_Xa (ROC20) correspond to leaves inoculated with *X. albilineans* and R0_CK (LCP 85–384) and S0_CK (ROC20) correspond to leaves inoculated with sterile liquid medium.

## Supplementary Materials

Supplementary materials can be found at https://www.mdpi.com/2076-2607/8/1/76/s1.
