# Supplementary material for: Identification of Differentially Expressed Proteins in Sugarcane in Response to Infection by Xanthomonas albilineans Using iTRAQ Quantitative Proteomics
Source: Microorganisms. 2020 Jan 3;8(1):76. doi: 10.3390/microorganisms8010076 (PMC7023244; doi:10.3390/microorganisms8010076)
Supplement: Supplementary file 1 [file microorganisms-08-00076-s001.zip › Supplemental files-20191216/Figure S1-20191214.pptx]

## Slide 1
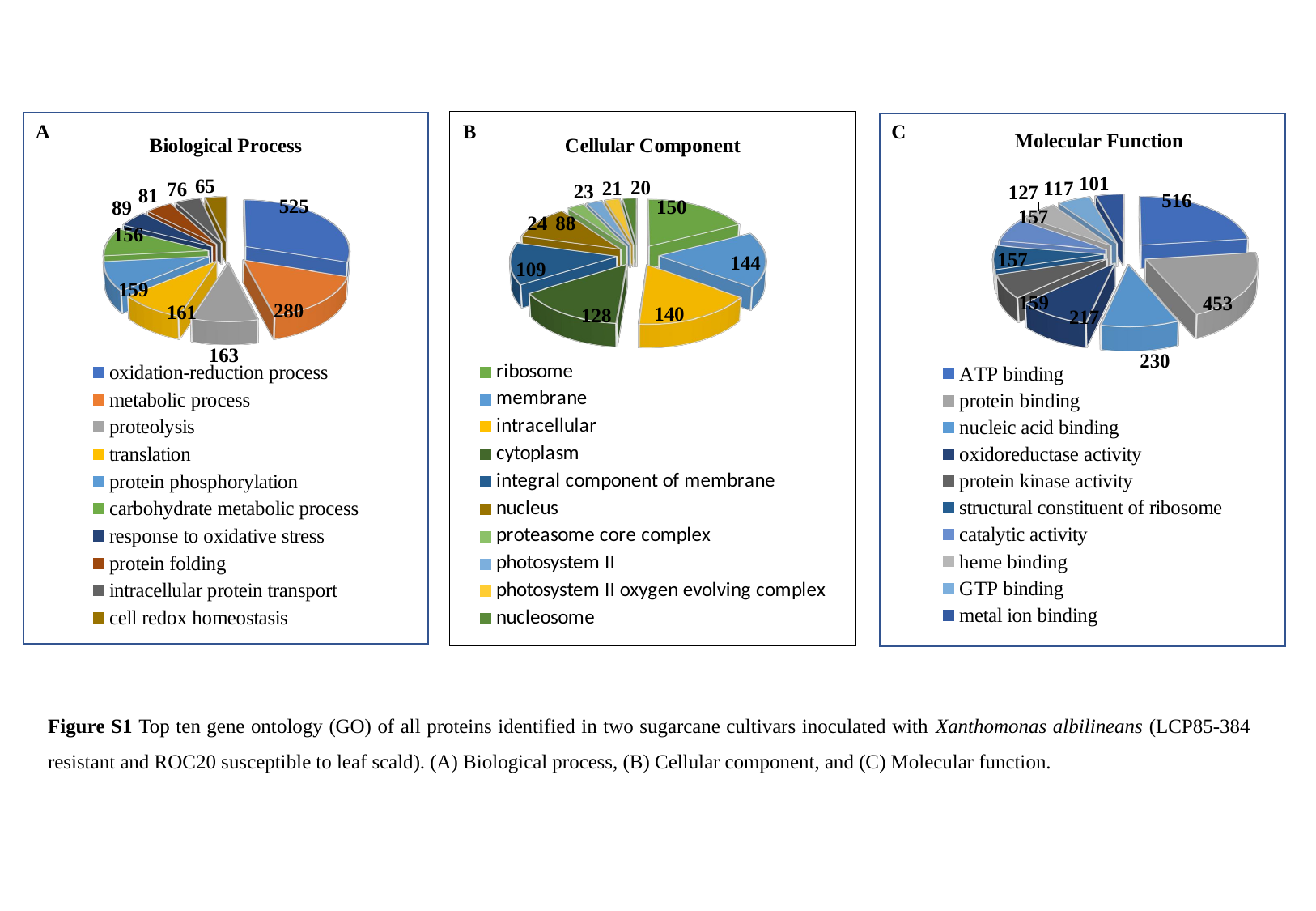

[unsupported chart]
[unsupported chart]
[unsupported chart]
B
A
C
Figure S1 Top ten gene ontology (GO) of all proteins identified in two sugarcane cultivars inoculated with Xanthomonas albilineans (LCP85-384 resistant and ROC20 susceptible to leaf scald). (A) Biological process, (B) Cellular component, and (C) Molecular function.
