# Supplementary material for: Identification of Differentially Expressed Proteins in Sugarcane in Response to Infection by Xanthomonas albilineans Using iTRAQ Quantitative Proteomics
Source: Microorganisms. 2020 Jan 3;8(1):76. doi: 10.3390/microorganisms8010076 (PMC7023244; doi:10.3390/microorganisms8010076)
Supplement: Supplementary file 1 [file microorganisms-08-00076-s001.zip › Supplemental files-20191216/Figure S2-20191216.pptx]

## Slide 1
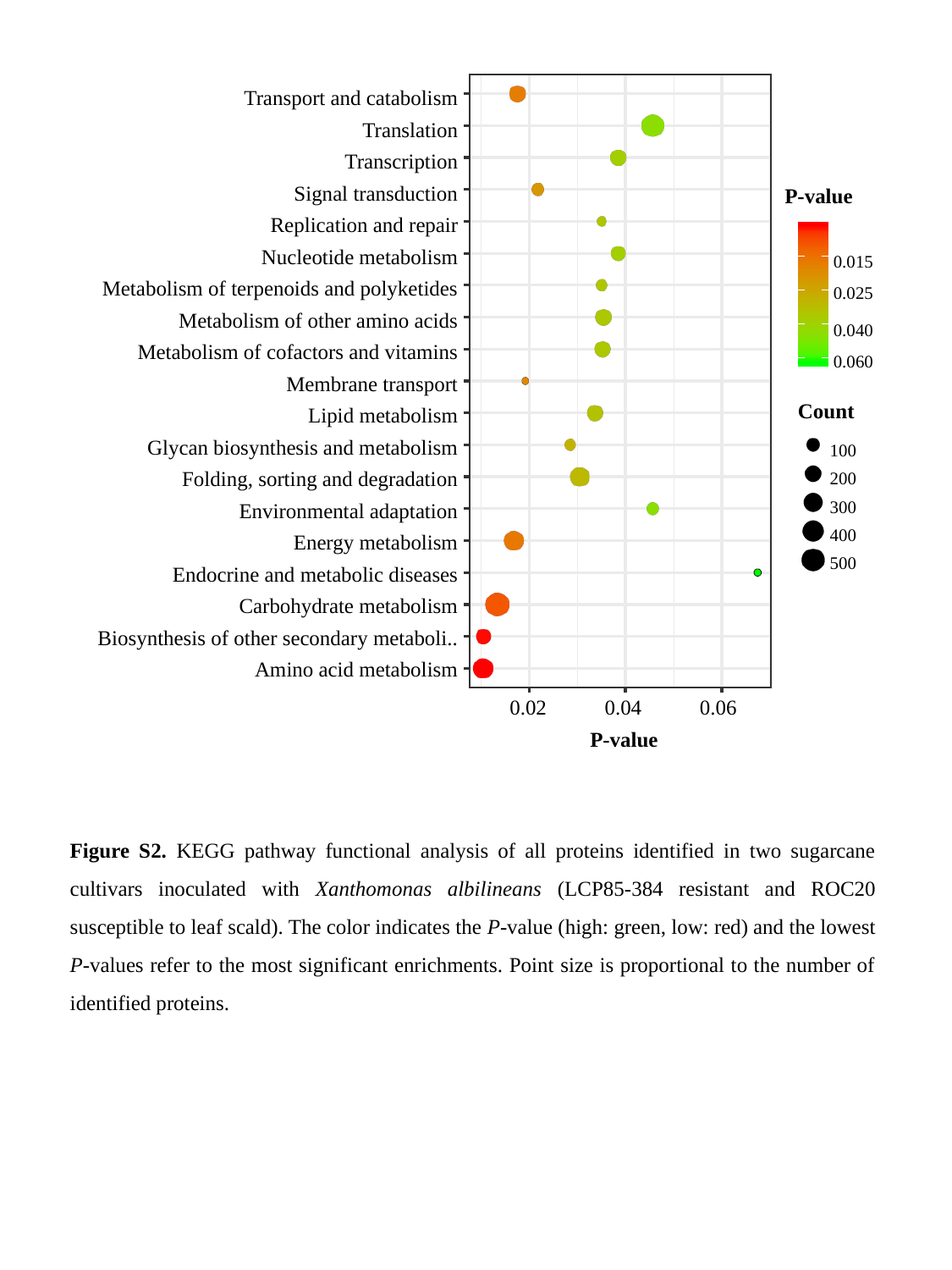

Transport and catabolism
Translation
Transcription
Signal transduction
Replication and repair
Nucleotide metabolism
Metabolism of terpenoids and polyketides
Metabolism of other amino acids
Metabolism of cofactors and vitamins
Membrane transport
Lipid metabolism
Glycan biosynthesis and metabolism
Folding, sorting and degradation
Environmental adaptation
Energy metabolism
Endocrine and metabolic diseases
Carbohydrate metabolism
Biosynthesis of other secondary metaboli..
Amino acid metabolism
P-value
0.015
0.025
0.040
0.060
Count
100
200
300
400
500
0.02 0.04 0.06
P-value
Figure S2. KEGG pathway functional analysis of all proteins identified in two sugarcane cultivars inoculated with Xanthomonas albilineans (LCP85-384 resistant and ROC20 susceptible to leaf scald). The color indicates the P-value (high: green, low: red) and the lowest P-values refer to the most significant enrichments. Point size is proportional to the number of identified proteins.
