# Supplementary material for: Identification of Differentially Expressed Proteins in Sugarcane in Response to Infection by Xanthomonas albilineans Using iTRAQ Quantitative Proteomics
Source: Microorganisms. 2020 Jan 3;8(1):76. doi: 10.3390/microorganisms8010076 (PMC7023244; doi:10.3390/microorganisms8010076)
Supplement: Supplementary file 1 [file microorganisms-08-00076-s001.zip › Supplemental files-20191216/Figure S3-20191214.pptx]

## Slide 1
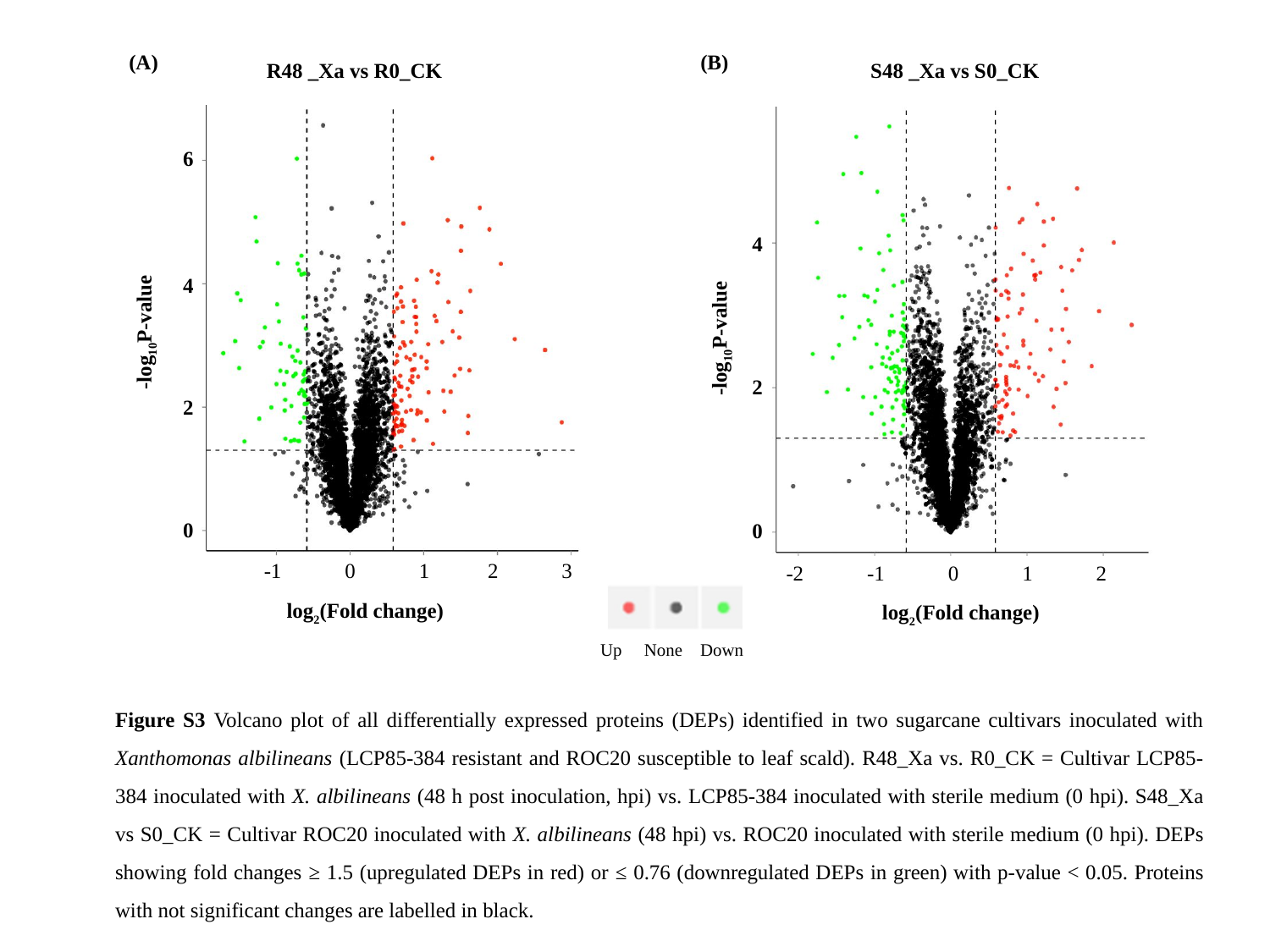

(A)
(B)
S48 _Xa vs S0_CK
R48 _Xa vs R0_CK
6
4
2
0
4
2
0
-log10P-value
-log10P-value
-1 0 1 2 3
-2 -1 0 1 2
 Up None Down
log2(Fold change)
log2(Fold change)
Figure S3 Volcano plot of all differentially expressed proteins (DEPs) identified in two sugarcane cultivars inoculated with Xanthomonas albilineans (LCP85-384 resistant and ROC20 susceptible to leaf scald). R48_Xa vs. R0_CK = Cultivar LCP85-384 inoculated with X. albilineans (48 h post inoculation, hpi) vs. LCP85-384 inoculated with sterile medium (0 hpi). S48_Xa vs S0_CK = Cultivar ROC20 inoculated with X. albilineans (48 hpi) vs. ROC20 inoculated with sterile medium (0 hpi). DEPs showing fold changes ≥ 1.5 (upregulated DEPs in red) or ≤ 0.76 (downregulated DEPs in green) with p-value < 0.05. Proteins with not significant changes are labelled in black.
