# Supplementary material for: Identification of Differentially Expressed Proteins in Sugarcane in Response to Infection by Xanthomonas albilineans Using iTRAQ Quantitative Proteomics
Source: Microorganisms. 2020 Jan 3;8(1):76. doi: 10.3390/microorganisms8010076 (PMC7023244; doi:10.3390/microorganisms8010076)
Supplement: Supplementary file 1 [file microorganisms-08-00076-s001.zip › Supplemental files-20191216/Figure S4-20191214.pptx]

## Slide 1
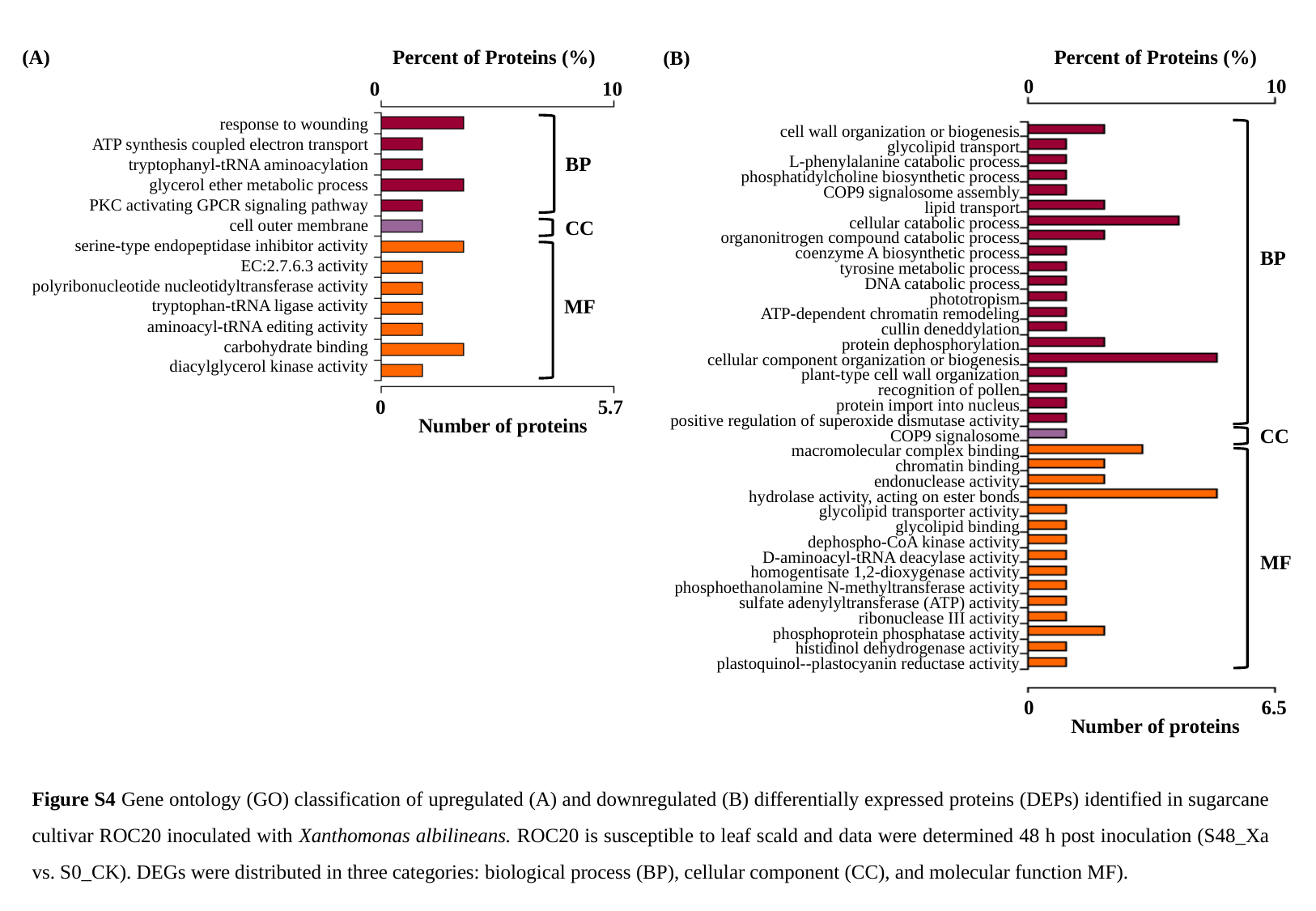

(A)
Percent of Proteins (%)
Percent of Proteins (%)
(B)
0 10
0 10
response to wounding
ATP synthesis coupled electron transport
tryptophanyl-tRNA aminoacylation
glycerol ether metabolic process
PKC activating GPCR signaling pathway
cell outer membrane
serine-type endopeptidase inhibitor activity
EC:2.7.6.3 activity
polyribonucleotide nucleotidyltransferase activity
tryptophan-tRNA ligase activity
aminoacyl-tRNA editing activity
carbohydrate binding
diacylglycerol kinase activity
cell wall organization or biogenesis
glycolipid transport
L-phenylalanine catabolic process
phosphatidylcholine biosynthetic process
COP9 signalosome assembly
lipid transport
cellular catabolic process
organonitrogen compound catabolic process
coenzyme A biosynthetic process
tyrosine metabolic process
DNA catabolic process
phototropism
ATP-dependent chromatin remodeling
cullin deneddylation
protein dephosphorylation
cellular component organization or biogenesis
plant-type cell wall organization
recognition of pollen
protein import into nucleus
positive regulation of superoxide dismutase activity
COP9 signalosome
macromolecular complex binding
chromatin binding
endonuclease activity
hydrolase activity, acting on ester bonds
glycolipid transporter activity
glycolipid binding
dephospho-CoA kinase activity
D-aminoacyl-tRNA deacylase activity
homogentisate 1,2-dioxygenase activity
phosphoethanolamine N-methyltransferase activity
sulfate adenylyltransferase (ATP) activity
ribonuclease III activity
phosphoprotein phosphatase activity
histidinol dehydrogenase activity
plastoquinol--plastocyanin reductase activity
BP
CC
BP
MF
0 5.7
Number of proteins
CC
MF
0 6.5
Number of proteins
Figure S4 Gene ontology (GO) classification of upregulated (A) and downregulated (B) differentially expressed proteins (DEPs) identified in sugarcane cultivar ROC20 inoculated with Xanthomonas albilineans. ROC20 is susceptible to leaf scald and data were determined 48 h post inoculation (S48_Xa vs. S0_CK). DEGs were distributed in three categories: biological process (BP), cellular component (CC), and molecular function MF).
