# Supplementary material for: Identification of Differentially Expressed Proteins in Sugarcane in Response to Infection by Xanthomonas albilineans Using iTRAQ Quantitative Proteomics
Source: Microorganisms. 2020 Jan 3;8(1):76. doi: 10.3390/microorganisms8010076 (PMC7023244; doi:10.3390/microorganisms8010076)
Supplement: Supplementary file 1 [file microorganisms-08-00076-s001.zip › Supplemental files-20191216/Table S1-20200102.docx]

Table S1 Genes and primers used in this study for qRT-PCR expression analysis.

| No. | Target gene ^a^ | Gene ID | Forward primer (5'-3') | Reverse primer (5'-3') | Tm (^o^C) | Amplicon size (bp) | Efficiency (%) |
| --- | --- | --- | --- | --- | --- | --- | --- |
| 1 | *psaA* | Cluster-4871.13787 | CGTCCGTCGGAACCAGAAGT | TCCAGATCCAAGTGGTAGTGTCG | 59 | 140 | 99.9 |
| 2 | *GAPC3* | Cluster-4871.143463 | GTCTCCAATGCTAGCTG | CAGTGATGGCATGAACA | 59 | 109 | 99.0 |
| 3 | *UGT* | Cluster-4871.235701 | ACGGAGAACATCGACATG | CTCGTCCATCTTCCACAA | 61 | 75 | 99.8 |
| 4 | *nsLTP* | Cluster-4871.183445 | TGCTCGATCTCACTACATTC | CCATGTCGACCTCTCTTG | 58 | 86 | 98.2 |
| 5 | *P450* | Cluster−4871.249909 | CACCATGATCCAAACATCTG | AGGAGAAGTGTTGAAGGATG | 60 | 196 | 99.6 |
| 6 | *AGO* | Cluster-4871.119964 | TGAGCAGCTGCGAGATGTGC | TGCAGGAGCTGGTGCATTCG | 59 | 199 | 99.7 |
| 7 | *UBA1* | Cluster-4871.278138 | CTCGGCGGCCTCCTACTCTA | CCAGCCATCTCCGCAACCTC | 60 | 143 | 99.4 |
| 8 | *GAPDH* |  | CACGGCCACTGGAAGCA | TCCTCAGGGTTCCTGATGCC | 58 | 110 | 101.0 |

^a^ psaA, photosystem I P700 apoprotein A1; GAPC3, cytosolic glyceroldehyde-3-phosphate dehydrogenase; UGT, UDP-glycosyltransferase; nsLTP, non-specific lipid transfer protein; P450, plant cytochrome P450 72A15; AGO*,* argonaute family protein; UBA1, ubiquitin-activating enzyme E1; GAPDH (reference gene), glyceraldehyde-3-phosphate dehydrogenase.
