# Supplementary material for: Identification of Differentially Expressed Proteins in Sugarcane in Response to Infection by Xanthomonas albilineans Using iTRAQ Quantitative Proteomics
Source: Microorganisms. 2020 Jan 3;8(1):76. doi: 10.3390/microorganisms8010076 (PMC7023244; doi:10.3390/microorganisms8010076)
Supplement: Supplementary file 1 [file microorganisms-08-00076-s001.zip › Supplemental files-20191216/Table S5-20191216.docx]

**Table S5** KEGG pathway enrichment analysis of differentially expressed proteins (DEPs) identified in sugarcane cultivars LCP85-384 (resistant to leaf scald) and ROC20 (susceptible to leaf scald) after inoculation with *Xanthomonas albilineans* ^a^

| **Pathway ID** | **Protein ID** | **Protein Description** | **LCP85-384** | | | **ROC20** | | |
| --- | --- | --- | --- | --- | --- | --- | --- | --- |
|  |  |  | **R0_CK** | **R48_Xa** | **log_2_FC** | **S0_CK** | **S48_Xa** | **log_2_FC** |
| ko00940 | Cluster-44733.0 | not available | 0.71 | 1.27 | **0.85** | 0.84 | 1.14 | NS |
| ko00940 | Cluster-4871.101959 | not available | 0.85 | 1.52 | **0.84** | 1.01 | 0.82 | NS |
| ko00940 | Cluster-4871.313888;orf1 | uncharacterized protein | 0.89 | 1.48 | **0.74** | 0.81 | 0.58 | NS |
| ko00940 | Cluster-4871.38543;orf1 | hypothetical protein | 0.70 | 1.18 | **0.76** | 1.08 | 0.73 | NS |
| ko00940 | Cluster-4871.393942;orf1 | hypothetical protein | 0.59 | 1.23 | **1.06** | 0.72 | 0.88 | NS |
| ko00940 | Cluster-4871.432784;orf1 | hypothetical protein | 0.76 | 1.15 | **0.59** | 0.64 | 0.88 | NS |
| ko00940 | Cluster-4871.85640;orf1 | hypothetical protein | 0.76 | 1.16 | **0.61** | 0.95 | 0.97 | NS |
| ko00940 | Cluster-4871.283132 | not available | 0.67 | 0.91 | NS | 0.71 | 1.12 | **0.67** |
| ko00940 | Cluster-4871.344626;orf1 | hypothetical protein | 1.00 | 1.41 | NS | 0.79 | 1.25 | **0.66** |
| ko04120 | Cluster-4871.231017;orf1 | hypothetical protein | 0.53 | 0.96 | NS | 0.57 | 0.97 | **0.76** |
| ko04120 | Cluster-4871.278138;orf1 | ubiquitin-activating enzyme E1 | 0.61 | 1.46 | **1.27** | 0.73 | 0.81 | NS |
| ko04120 | Cluster-4871.291736 | not available | 1.82 | 0.89 | **-1.04** | 1.37 | 1.01 | NS |
| ko04120 | Cluster-4871.173568;orf1 | not available | 0.79 | 0.93 | NS | 0.50 | 1.09 | **1.14** |
| ko00195 | Cluster-4871.13787;orf1 | photosystem I P700 apoprotein A1 (chloroplast) | 0.58 | 1.04 | **0.83** | 0.69 | 0.92 | NS |
| ko00195 | Cluster-4871.288803 | not available | 1.33 | 0.76 | **-0.80** | 1.15 | 1.08 | NS |
| ko00195 | Cluster-4871.241008 | not available | 1.53 | 1.30 | NS | 0.84 | 0.56 | **-0.60** |
| ko00195 | Cluster-4871.237971;orf1 | hypothetical protein | 1.33 | 0.72 | **-0.89** | 0.98 | 1.11 | NS |
| ko00480 | Cluster-4871.226640;orf1 | predicted protein | 1.28 | 0.78 | **-0.71** | 1.00 | 0.82 | NS |
| ko00480 | Cluster-4871.341601 | not available | 0.65 | 1.02 | **0.66** | 1.23 | 0.97 | NS |
| ko00480 | Cluster-4871.292606;orf1 | hypothetical protein | 0.75 | 1.21 | **0.68** | 0.80 | 0.82 | NS |
| ko00196 | Cluster-4871.237470;orf1 | unknown [*Zea mays*] | 1.20 | 0.79 | **-0.60** | 1.03 | 1.14 | NS |
| ko00196 | Cluster-4871.238350;orf1 | hypothetical protein | 1.21 | 0.78 | **-0.63** | 1.11 | 1.36 | NS |
| ko04016 | Cluster-4871.243737;orf1 | serine/threonine-protein kinase SAPK2-like isoform | 0.48 | 1.06 | **1.13** | 0.44 | 1.08 | **1.31** |
| ko00908 | Cluster-4871.308103;orf1 | cis-zeatin O-glucosyltransferase | 1.00 | 0.37 | **-1.43** | 1.04 | 0.43 | **-1.26** |
| ko00770 | Cluster-4871.148300;orf1 | hypothetical protein | 1.06 | 0.37 | **-1.51** | 1.01 | 0.38 | **-1.39** |
| Unavailable | Cluster-4871.119964 | argonaute family protein | 0.49 | 1.01 | **1.04** | 0.49 | 0.86 | **0.82** |
| Unavailable | Cluster-4871.143463 | cytosolic glyceroldehyde-3-phosphate dehydrogenase GAPC3 | 0.45 | 1.13 | **1.33** | 0.43 | 1.01 | **1.22** |
| Unavailable | Cluster-4871.183059;orf1 | disease resistance protein RPM1 | 0.75 | 1.40 | **0.91** | 0.23 | 0.65 | **1.46** |
| Unavailable | Cluster-4871.183445;orf1 | Non-specific lipid-transfer protein | 0.91 | 1.91 | **1.06** | 0.78 | 0.39 | **-0.99** |
| Unavailable | Cluster-4871.235701 | UDP-glycosyltransferase | 0.36 | 1.10 | **1.62** | 0.27 | 0.81 | **1.59** |
| Unavailable | Cluster-4871.245933;orf1 | Ras-related protein Rab-5C | 0.64 | 1.04 | **0.70** | 0.63 | 0.95 | **0.59** |
| Unavailable | Cluster-4871.249909;orf1 | cytochrome P450 72A15 | 0.29 | 0.89 | **1.60** | 0.27 | 0.75 | **1.48** |
| Unavailable | Cluster-4871.305904 | amidophosphoribosyltransferase, | 0.48 | 1.10 | **1.20** | 0.48 | 0.98 | **1.04** |
| Unavailable | Cluster-4871.316854;orf2 | mitochondrial uncoupling protein 2 | 0.50 | 0.92 | **0.87** | 0.37 | 0.77 | **1.07** |
| Unavailable | Cluster-4871.340096;orf1 | adenylate cyclase associated (CAP) N terminal | 0.99 | 1.55 | **0.65** | 0.46 | 0.93 | **1.01** |

**^a^** ko00940, Phenylpropanoid biosynthesis; ko04120, Ubiquitin mediated proteolysis; ko00195, Photosynthesis; ko00480, Glutathione metabolism; ko04016, Mitogen-activated protein kinase (MAPK) signaling pathway-plant; ko00196, Photosynthesis-antenna proteins; ko00908, Zeatin biosynthesis; ko00770, Pantothenate and CoA biosynthesis. R0_CK = cultivar LCP85-384 inoculated with sterile liquid medium, R48_Xa = cultivar LCP85-384 inoculated with *X. albilineans*, S0_CK = cultivar ROC20 inoculated with sterile liquid medium, S48_Xa = cultivar ROC20LCP85-384 inoculated with *X. albilineans.* The values of log_2_FC of differentially expressed proteins (DEPs) shown in bold were significant at *p* = 0.05; NS means non-significant.
